# The Influence of VE-Cadherin on Adhesion and Incorporation of Breast Cancer Cells into Vascular Endothelium

**DOI:** 10.3390/ijms22116049

**Published:** 2021-06-03

**Authors:** Thomas Brock, Elisabeth Boudriot, Anke Klawitter, Marianne Großer, Trang T. P. Nguyen, Sindy Giebe, Erik Klapproth, Achim Temme, Ali El-Armouche, Georg Breier

**Affiliations:** 1 Division of Medical Biology, Department of Psychiatry and Psychotherapy, Faculty of Medicine Carl Gustav Carus, TU Dresden, 01307 Dresden, Germany; thomas.brock@tu-dresden.de (T.B.); elisabeth.boudriot@tu-dresden.de (E.B.); anke.klawitter@uniklinikum-dresden.de (A.K.); trang.nguyen@uniklinikum-dresden.de (T.T.P.N.); 2 Institute of Pharmacology and Toxicology, Faculty of Medicine Carl Gustav Carus, TU Dresden, 01307 Dresden, Germany; erik.klapproth@tu-dresden.de (E.K.); ali.el-armouche@tu-dresden.de (A.E.-A.); 3 Institute of Pathology, University Hospital, TU Dresden, 01307 Dresden, Germany; marianne.grosser@uniklinikum-dresden.de; 4 Division of Vascular Endothelium and Microcirculation, Department of Medicine III, Faculty of Medicine Carl Gustav Carus, TU Dresden, 01307 Dresden, Germany; sindy.giebe@tu-dresden.de; 5 Division of Experimental Neurosurgery/Tumor Immunology, Department of Neurosurgery, Faculty of Medicine Carl Gustav Carus, TU Dresden, 01307 Dresden, Germany; achim.temme@uniklinikum-dresden.de

**Keywords:** metastasis, transendothelial migration, VE-cadherin, breast cancer

## Abstract

During metastasis, cancer cells that originate from the primary tumor circulate in the bloodstream, extravasate, and form micrometastases at distant locations. Several lines of evidence suggest that specific interactions between cancer cells and endothelial cells, in particular tumor cell adhesion to the endothelium and transendothelial migration, play a crucial role in extravasation. Here we have studied the role of vascular endothelial (VE)-cadherin which is expressed aberrantly by breast cancer cells and might promote such interactions. By comparing different human breast cancer cell lines, we observed that the number of cancer cells that adhered to endothelium correlated with VE-cadherin expression levels. VE-cadherin silencing experiments confirmed that VE-cadherin enhances cancer cell adhesion to endothelial cells. However, in contrast, the number of cancer cells that incorporated into the endothelium was not dependent on VE-cadherin. Thus, it appears that cancer cell adhesion and incorporation are distinct processes that are governed by different molecular mechanisms. When cancer cells incorporated into the endothelial monolayer, they formed VE-cadherin positive contacts with endothelial cells. On the other hand, we also observed tumor cells that had displaced endothelial cells, reflecting either different modes of incorporation, or a temporal sequence where cancer cells first form contact with endothelial cells and then displace them to facilitate transmigration. Taken together, these results show that VE-cadherin promotes the adhesion of breast cancer cells to the endothelium and is involved in the initial phase of incorporation, but not their transmigration. Thus, VE-cadherin might be of relevance for therapeutic strategies aiming at preventing the metastatic spread of breast cancer cells.

## 1. Introduction

Breast cancer is the most frequently diagnosed cancer in women and a leading cause of cancer mortality [1]. Triple negative breast cancer, which is characterized by the lack of expression of the estrogen and progesterone receptors and by amplification of human epidermal growth factor receptor 2 (HER2)/Neu, offers a particularly poor prognosis with limited therapeutic options and thus urgently requires new treatment approaches [2]. Given that metastases, rather than primary tumors, are responsible for the majority of cancer deaths [3], it is important to understand the cellular and molecular mechanisms leading to the metastatic dissemination of cancer cells.

Metastasis occurs when invasive tumor cells intravasate into blood or lymph vessels, circulate, extravasate, and settle in anatomically distant locations. The vascular endothelium represents a barrier for invasive tumor cells and thus limits the metastatic dissemination of cancer cells via the bloodstream or the lymphatics [4]. The barrier function of the endothelium is dependent on vascular endothelial (VE-) cadherin, which forms homophilic contacts at endothelial adherens junctions and thus connects the cytoskeletal actin filaments of neighboring cells [5,6]. Yet, the endothelial barrier is often compromised in tumors, as tumor vessels are leaky as a result of the activity of vascular permeability factors such as vascular endothelial growth factor (VEGF), which are abundantly expressed by tumor and stromal cells [7,8]. This facilitates the intravasation of invasive cancer cells. In contrast, at the site of extravasation, tumor cells must cross the intact endothelium and the underlying basement membrane. Adhesion of cancer cells to the endothelium and the subsequent transmigration of tumor cells through the endothelium (Transendothelial migration, or TEM) at the site of extravasation represent critical steps in metastasis. The interaction of tumor cells with endothelial cells can therefore be considered as a key event in metastasis. Yet, the exact mechanisms of extravasation are only incompletely characterized [9]. Stable cancer cell-endothelial cell adhesion may be mediated by various adhesion receptors, including neural (N-) cadherin, certain integrins, cluster of differentiation (CD)44 and mucin-1 (MUC1) [9,10]. TEM occurs after adhesion and involves the incorporation of tumor cells into the endothelial layer and the subsequent liberation [9]. Tumor cells may cross the endothelial cell barrier in different ways, with paracellular transmigration, rather than transcellular migration being the most common mechanism [9,11]. Paracellular migration requires the disruption of endothelial adherens junctions formed by VE-cadherin.

We have previously found that tumor cells in murine and human breast cancer may express aberrantly VE-cadherin [12,13] whose expression is normally restricted to the vascular endothelium [6,9,14]. In a murine breast cancer model, VE-cadherin promoted tumor growth in vivo and tumor cell adhesion to vascular endothelial cells in vitro [12,13]. Moreover, VE-cadherin was identified as a prognostic marker for metastasis in human breast cancer patients, further supporting a role for VE-cadherin in breast cancer progression [15]. However, the exact functions of VE-cadherin in aggressive human breast cancer cells remained unclear. Circumstantial evidence based on the forced overexpression of VE-cadherin in MCF-7 and MDA-MB-231 breast cancer cells supports the view that VE-cadherin promotes tumor-endothelial interactions also in human cells [16]. This led us to hypothesize that aberrant VE-cadherin expression on human mammary carcinoma cells might, through the formation of contacts with endothelial cells, facilitate their adhesion and potentially their transmigration through the endothelium.

Therefore, in this study, we have identified and characterized human breast cancer cell lines that show endogenous VE-cadherin expression. Triple negative BT20 and SUM149PT human breast cancer cells expressed significant levels of VE-cadherin mRNA and protein. The potential of breast cancer cells to adhere to human endothelial cells correlated with the expression level of VE-cadherin, and RNA interference experiments showed that VE-cadherin downregulation reduced the number of breast cancer cells that adhered to endothelial cells, but did not completely inhibit adhesion. Together, the results suggest that aberrantly expressed VE-cadherin enhances the adhesion of human breast cancer cells to endothelial cells, but does not necessarily promote their incorporation into the endothelial layer. The results further elucidate the role of VE-cadherin in breast cancer progression and the mechanisms of tumor cell-endothelial cell interactions.

## 2. Results

### 2.1. BT20 und SUM149 Cells Differentially Express VE-, N- and E-Cadherin

**Figure 1:**
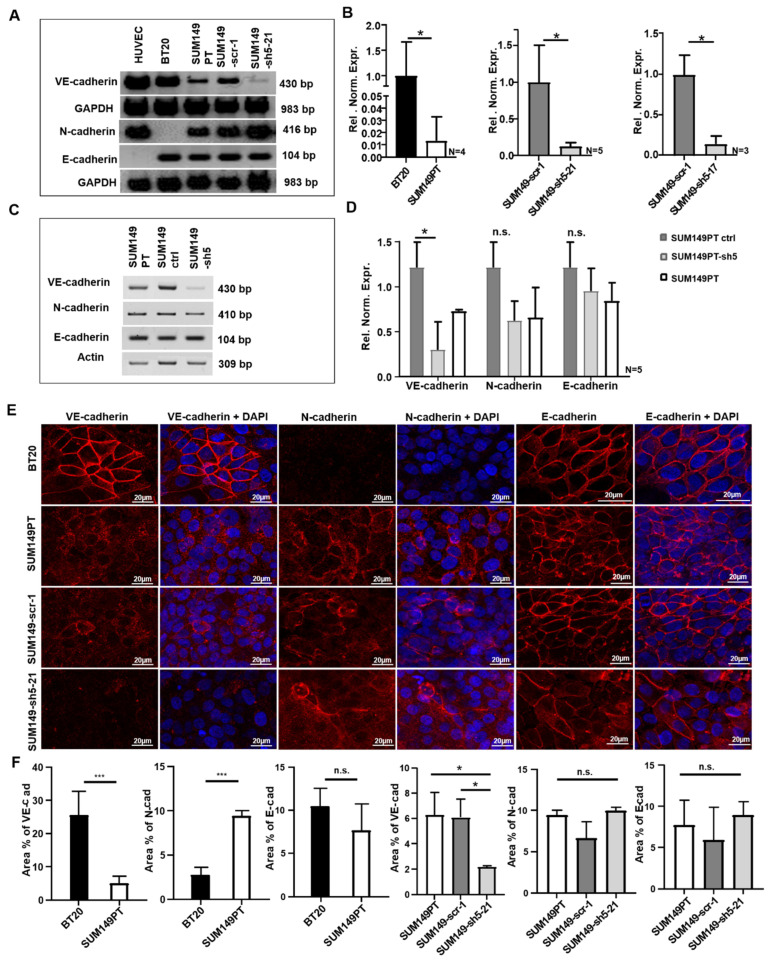
Expression of VE-, N- and E-cadherin in BT20, SUM149PT, SUM149-scr-1, SUM149-sh5-21, SUM149 ctrl and SUM149-sh5 cells, as detected by RT-PCR, qRT-PCR and immunofluorescence analysis. (**A**) VE-, N- and E-cadherin mRNA expression by RT-PCR. (**B**) Quantification of VE-cadherin mRNA by qRT-PCR. Relative normalized expression (Rel. Norm. Expr.). (**C**) VE-, N- and E-cadherin mRNA expression as observed by RT-PCR. (**D**) Quantification of VE-, N and E-cadherin mRNA by qRT-PCR. Relative normalized expression (Rel. Norm. Expr.). (**E**) Immunofluorescence analysis of VE- N-, and E-cadherin. Nuclear staining with DAPI, 40x magnification (**F**) Area in percent (area in %) positive for VE-, N-and E-cadherin immunofluorescence. For calculation, two to five random spots of the images were used. Bars represent standard deviation; n.s. not significant, * *p* < 0.5, *** *p* ≤ 0.001; statistical analysis was conducted using unpaired Welch’s *t*-tests.

Various human breast cancer cell lines were examined by RT-PCR for the expression of VE-, N- and E-cadherin. Two triple negative cell lines, BT20 and SUM149PT, which showed different expression levels of VE-cadherin mRNA and protein, were selected for further analysis. BT20 cells expressed abundant VE-cadherin mRNA, whereas SUM149PT cells showed a somewhat weaker expression (Figure 1A,B). Both cell lines also expressed E-cadherin mRNA. N-cadherin mRNA was readily detected in SUM149PT but not in BT20 cells, reflecting low expression levels in the latter cell line (Figure 1A). Because one of our aims was to study the potential interplay between these three classical cadherins, as previously performed in the murine system [13], we chose SUM149PT cells for VE-cadherin knockdown experiments.

VE-cadherin deficient SUM149PT cell clones were generated by transduction with recombinant lentiviruses encoding VE-cadherin specific shRNA. Of the resulting single cell clones, SUM149-sh5-21 and SUM149-sh5-17 showed a strong reduction in VE-cadherin mRNA expression (Figure 1A). SUM149PT control clones which contained an unspecific scrambled shRNA sequence, SUM149-scr-1, were also generated. The qRT-PCR analysis revealed 87% or 86% reduction in VE-cadherin mRNA expression for SUM149-sh5-21 and SUM149-sh5-17, respectively, compared to SUM149-scr-1 (Figure 1B). SUM149PT, SUM149-scr-1 cells, and SUM149-sh5-21 cells showed similar levels of N- and E-cadherin mRNA (Figure 1A). 

To complement the analysis of single cell clones, we also generated a SUM149PT VE-cadherin knockdown cell line by lentiviral transduction of a whole culture of tumor cells. The resulting cell line thus contained a mixture of individually transduced cells. SUM149PT ctrl cells were generated by transduction of cells with empty vector. SUM149-sh5 cells expressed less VE-cadherin mRNA compared to SUM149 ctrl control cells. The VE-cadherin knockdown efficiency was 69.9% as verified by qRT-PCR. Both cell lines showed similar expression levels of N- or E-cadherin mRNA (Figure 1C,D).

### 2.2. BT20, SUM149PT, SUM149-Scr-1 and SUM149-sh5-21 Cells Express VE-, E- and N-Cadherin on the Cell Surface

Immunofluorescence analysis was performed to detect the localization of cadherin proteins. In BT20 cells, VE-cadherin was localized predominantly at the cell-cell-contacts (Figure 1E), consistent with localization at the cell surface. In contrast, a more diffuse distribution of VE-cadherin immunoreactivity was observed in SUM149PT and SUM149-scr-1 cells. The VE-cadherin deficient SUM149-sh5-21 cells showed no VE-cadherin expression, as expected (Figure 1E). Consistent with the result of the RT-PCR analysis, very low N-cadherin immunoreactivity was observed for BT20 cells. SUM149PT, SUM149-scr-1 and SUM149-sh5-21 cells, however, showed N-cadherin staining both at the cell periphery and scattered over the cell (Figure 1E). No significant difference in the pattern of N-cadherin expression was observed between SUM149-scr-1 and SUM149-sh5-21 cells, indicating that VE-cadherin expression does not influence the subcellular localization of N-cadherin in SUM149PT cells. In BT20 cells as well as in SUM149PT, SUM149-scr-1 and SUM149-sh5-21 cells, E-cadherin was localized predominantly at the cell membrane (Figure 1E).

Next, quantification of the immunofluorescence staining was performed (Figure 1F). BT20 and SUM149PT cells differed in VE-cadherin (25.5% vs. 4.9% area positive for VE-cadherin) and N-cadherin expression (2.7% vs. 9.4% area positive for N-cadherin), but not significantly in E-cadherin protein expression (10.5% vs. 7%. area positive for E-cadherin). As compared to SUM149PT or SUM149-scr-1 control cells, SUM149-sh5-21 cells showed reduced VE-cadherin expression (6.3% vs. 6.1% vs. 2.2% VE-cadherin positive area, respectively) but comparable levels of N-cadherin (9.4% vs. 6.6% vs. 9.9% N-cadherin positive area) and E-cadherin protein (7.7% vs. 5.9% vs. 8.9% E-cadherin positive area).

### 2.3. VE-Cadherin Enhances Breast Cancer Cell Adhesion to HUVEC Endothelium

**Figure 2:**
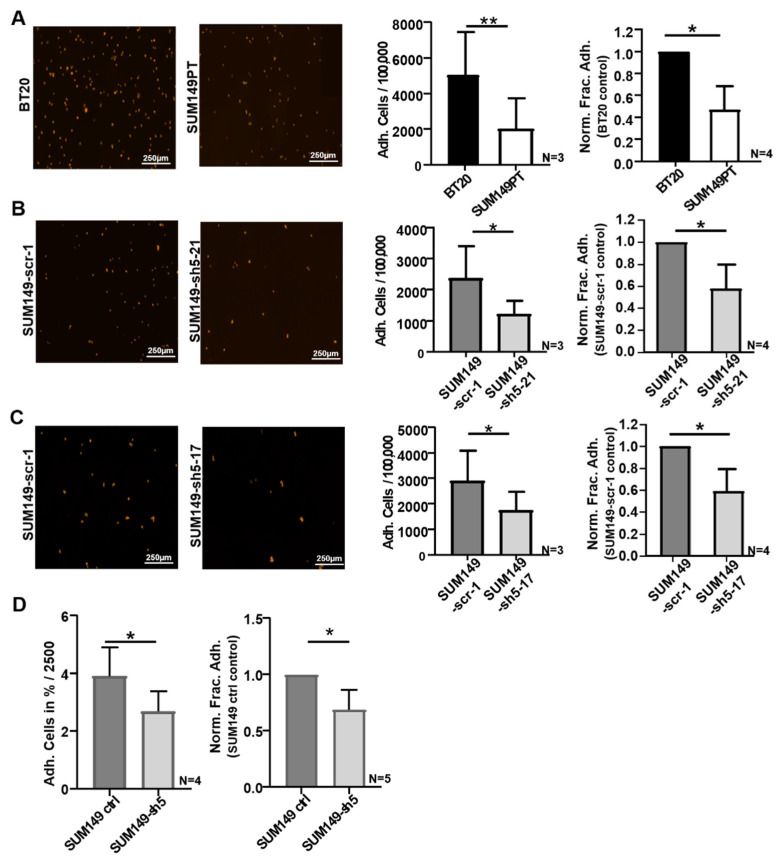
VE-cadherin influences the adhesion of BT20, and SUM149 cells to HUVEC. (**A**–**C**) Identical cell numbers (100,000) of fluorescently labelled tumor cells were incubated on HUVEC monolayers for 30 min. Immunofluorescence images as used for cell counting by Fiji software (left panels); Number of tumor cells adherent to HUVEC of the 100,000 tumor cells seeded (Adh. Cells/100,000); Normalized Fraction Adhesion (Norm. Frac. Adh.) to control (right panels). (**D**) Identical numbers (2500) of fluorescently labelled tumor cells were incubated on HUVEC monolayers for 60 min. Fluorescence intensity measurement was performed. Tumor cell number correlated to fluorescence intensity and was determined by RFU standard curve (Supplementary Figure S1). Adherent tumor cells in percent of the 2500 tumor cells seeded (Adh. Cells in %/2500) and the Normalized Fraction Adhesion (Norm. Frac. Adh.) to control; Bars represents standard deviation; * *p* ≤ 0,05; ** *p* ≤ 0.01; statistical analysis was conducted using unpaired Welch’s *t*-tests.

Adhesion of tumor cells to the endothelium of blood vessels is one of the major requirements of TEM during hematogenic metastasis. In order to investigate the potential influence of VE-cadherin on this interaction, adhesion experiments were carried out with BT20 and SUM149PT cells, as well as with SUM149-scr-1, SUM149-sh5-21 and SUM149-sh5-17 cells. These assays were analyzed by automated microscopical counting of fluorescently labeled tumor cells (Figure 2A–C). This technique allowed us to directly count the number of adherent cells. Of 100,000 tumor cells seeded, on average 5.0% of BT20 cells (5023 cells) and 2.0% SUM149PT cells (2016 cells) were attached to the endothelium after 30 min (Figure 2A). Thus, a significantly higher number of BT20 cells adhered to HUVEC as compared to SUM149PT cells. On average, the number of SUM149PT cells was 53% lower in comparison to BT20 cells (Figure 2A). Because BT20 cells express higher VE-cadherin levels than SUM149PT cells, this result might suggest that VE-cadherin promotes adhesion of cancer cells to endothelial cells.

To verify the potential influence of VE-cadherin expression on the adhesion of tumor cells to endothelial cells, we performed adhesion experiments with VE-cadherin deficient cell clones, SUM149-sh5-21 and SUM149-sh5-17. Out of 100,000 tumor cells seeded, on average 2.3% or 2.8% of SUM149-scr-1 cells (2360 and 2893 cells, respectively), 1.2% SUM149-sh5-21 cells (1208 cells) and 1.7% SUM149-sh5-17 cells (1747 cells) adhered to the HUVEC monolayer (Figure 2B,C). As compared to the control cells (SUM149-scr-1), a significantly lower number of VE-cadherin knockdown cells adhered to HUVEC monolayers. On average, a 41% and 42% reduction of adhesion to HUVEC compared to SUM149-scr-1 cells was observed for the knockdown cell clones, respectively (Figure 2B,C).

To complement the analysis of single cell clones, we also used the SUM149PT VE-cadherin knockdown cell line, SUM149-sh5, which contained a mixture of individually transduced tumor cells (see Figure 1C,D), and used an independent technique for quantification. SUM149PT ctrl cells were used as a control. Tumor cells were fluorescently labeled, and adhesion to HUVEC endothelial cells was determined by measuring the fluorescence intensity of each well using a microplate reader, as described in Material and Methods. Fluorescence intensity was shown to correlate with tumor cell number, and the number of bound tumor cells was calculated with an RFU standard curve (Supplementary Figure S1). Of 2500 tumor cells seeded, 3.9% of the SUM149 ctrl cells and 2.7% of the VE-cadherin deficient SUM149-sh5 cells, respectively, adhered to the endothelium. VE-cadherin deficient SUM149-sh5 cells thus showed a 31.2% reduced adhesion to HUVEC as compared to SUM149 ctrl cells (Figure 2D). The primary data of the adhesion experiments are displayed in Supplementary Tables S1–S3. Taken together, the data show that VE-cadherin enhances the adhesion of human breast cancer cells to HUVEC endothelial cells.

### 2.4. BT20, SUM149PT, and VE-Cadherin Deficient SUM149PT Cells Incorporate into HUVEC Endothelium

**Figure 3:**
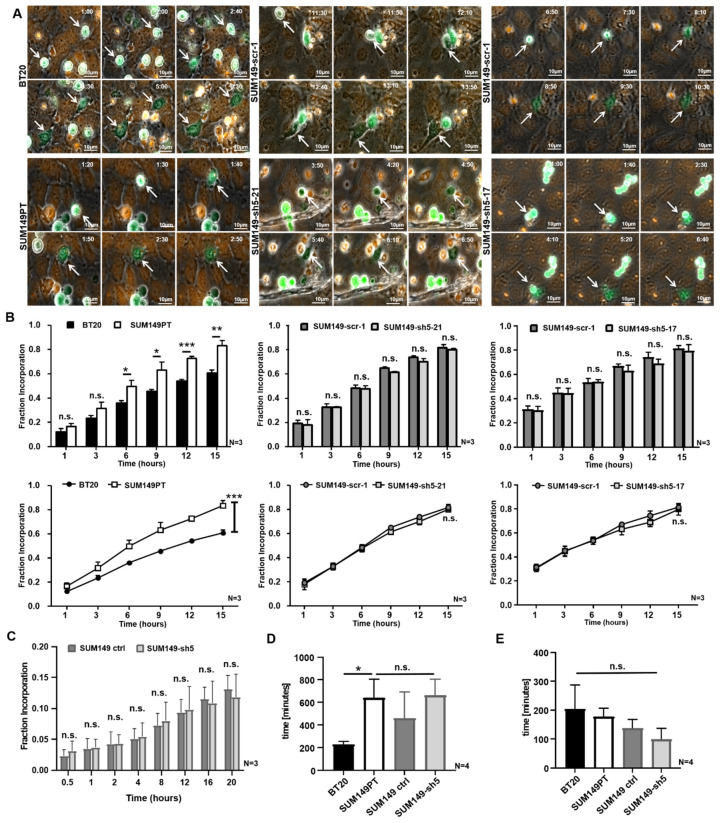
The incorporation of SUM149 cells into the HUVEC monolayer is VE-cadherin independent. 50,000 tumor cells (green) were stained with CellTracker Green and placed on a confluent HUVEC monolayer (orange), fluorescently labeled with CellTracker Orange. The incorporation was recorded over 15 h with a microscope focused on the HUVEC monolayer. (**A**) The BT20, SUM149PT, SUM149-scr-1, SUM149-sh5-21 and SUM149-sh5-17 cells (white arrows) moved into the focus plane of HUVEC and incorporated into the endothelium. 40x magnification. Time in h:min after the beginning of the experiment is indicated; (**B**) Fraction of Incorporation at 1, 3, 6, 9, 12 or 15 h after the start of experiment; (**C**) 20,000 tumor cells were placed on a HUVEC monolayer, and their incorporation was recorded over 20 h with microscope focus on the HUVEC plane. Fraction of Incorporation 0.5, 1, 2, 4, 8, 12, 16 and 20 h after the start of the experiment; (**D**) Incorporation inception times do not depend on VE-cadherin as analyzed by live cell imaging (**E**) Duration of incorporation is VE-cadherin independent as calculated by live cell imaging. Bars represent standard deviation; n.s. not significant, * *p* ≤ 0.05; ** *p* ≤ 0.01; *** *p* ≤ 0.001; statistical analysis was conducted using one-way and two-way ANOVA.

Transient incorporation into the endothelium is thought to be a crucial step in the course of TEM. To investigate whether VE-cadherin might play a role in this process, we used live cell imaging to monitor how BT20, SUM149PT, SUM149-scr-1, SUM149-sh5-21 and SUM149-sh5-17 cells incorporate into a confluent HUVEC monolayer. First, we compared the incorporation behavior of BT20 and SUM149 cells. Next, the VE-cadherin expressing control cells, SUM149-scr-1 were compared with the VE-cadherin deficient cell clones, SUM149-sh5-21 and SUM149-sh5-17, to analyze the potential influence of VE-cadherin on the incorporation. Live cell imaging of single cell movements revealed that tumor cells initially moved on top of the HUVEC monolayer and only incorporated over time. The focus of the live cell images was set to the HUVEC plane and thus, the tumor cells were only sharply imaged after they had integrated into the HUVEC monolayer. As the tumor cells moved into the focus plane of the HUVEC, their phenotype changed from a rounded to a rather stretched and asymmetrical morphology (Figure 3A and Supplementary Video S1–S5). These two criteria were defined as characteristics of incorporation. Furthermore, we observed that the incorporation process of BT20, SUM149PT, SUM149-scr-1, SUM149-sh5-21, and SUM149 sh5-17 cells were not uniform. For every tumor cell line both early (1 h after adhesion) and late (11.5 h after adhesion) incorporations could be seen, some of which were very fast and some very slow (Figure 3A).

### 2.5. SUM149PT Cells Incorporate More Frequently Than BT20 Cells into HUVEC Endothelium

Next we investigated whether there was a quantitative difference in the number of incorporating BT20 and SUM149PT cells. To this end, the proportion of incorporated cells was determined at different time points after seeding of the tumor cells. The results showed that SUM149PT cells incorporated more often than BT20 cells into the HUVEC monolayer. After 1 h or 3 h, there was no significant difference in the proportion of integrated tumor cells (Figure 3B). Later on, a higher proportion of SUM149PT cells had incorporated at all investigated time points. At 15 h after the beginning of the experiment, an average of 60.7% of the BT20 cells and 83.3% of the SUM149PT cells had been incorporated in the HUVEC. The result of the two-way ANOVA test indicates that the time (p ≤ 0.001), as well as the tumor cell lines (BT20 or SUM149PT), have an effect on incorporation. A significantly (*p* ≤ 0.006) higher proportion of SUM149PT cells had integrated compared to the BT20 cells at the end of the experiment (Figure 3B). Considering that BT20 cells expressed considerably higher VE-cadherin levels than SUM149PT cells, this result might indicate that VE-cadherin does not enhance tumor cell incorporation into the HUVEC monolayer. A table with the calculations of Fraction Incorporation is shown in Supplementary Table S4.

### 2.6. VE-Cadherin Has no Quantitative Influence on the Incorporation Rate of Breast Cancer Cells 

To verify this assumption, we quantified the incorporation of SUM149-scr-1 control cells to the corresponding VE-cadherin knockdown SUM149-sh5-21 and SUM149 sh5-17 cells in the same way. The comparison of these cells revealed no significant difference in the proportion of incorporated cells at the time points investigated. At 15 h after the onset of adhesion, an average of 82% or 81% of the SUM149 scr 1 cells had incorporated into the endothelium whereas SUM149-sh5-21 and SUM149 sh5-17 cells each showed an 80% proportion of incorporated cells. As observed for BT20 and SUM149PT cells, the two-way ANOVA test revealed that the incorporation of SUM149-scr-1, SUM149 sh5 21 and SUM149 sh5-17 cells was a time-dependent process. The proportion of incorporated cells increased significantly over time (*p* ≤ 0.001), but there was no quantitative difference between SUM149-scr-1, SUM149 sh5-21 and SUM149 sh5-17 cells (Figure 3B).

Similarly, the experiments performed with SUM149 ctrl and SUM149-sh5 cells did not reveal differences in the fraction of incorporation from 0.5 h up to 20 h after the start of the assay (Figure 3C). Taken together, these results support the conclusion that VE-cadherin expressed by breast cancer cells had no quantitative influence on the incorporation rate of tumor cells. A table with the calculations of Fraction Incorporation from experiments is shown in Supplementary Table S5.

### 2.7. VE-Cadherin Has No Influence on Incorporation Inception Time or Duration 

To determine whether VE-cadherin might influence inception time or duration of incorporation into the endothelial layer, we extended the live cell imaging analysis for BT20, SUM149PT, SUM149 ctrl and the VE-cadherin deficient SUM149-sh5 cell lines. The results show that incorporation inception times vary among different tumor cell lines (BT20 vs. SUM149PT) but do not depend on VE-cadherin expression (SUM149 ctrl vs. SUM149-sh5). BT20 cells began to incorporate on average after 228 min. This was significantly earlier than SUM149PT cells which showed an average incorporation inception time of 640 min. The incorporation inception times for VE-cadherin knockdown SUM149-sh5 and their control cells did not differ significantly at 662 min and 460 min, respectively (Figure 3D). The duration of the incorporation process did not vary significantly between the different tumor cell lines and was approximately 152 min. The tumor cells, as mentioned above, displayed a wide range of time spans, e.g., from 30 min to 770 min for BT20 cells (204 min on average). SUM149PT-sh5 cells displayed the shortest incorporation time span at 10 min to 550 min (on average 98 min) (Figure 3E). The incorporation duration time did also not differ between SUM149PT and SUM149 ctrl cells (177 min vs. 137 min). These results demonstrate that VE-cadherin had no influence on inception time or duration of incorporation.

### 2.8. N-Cadherin Influences Adhesion and Incorporation of SUM149 Cells

Furthermore, we investigated whether N-cadherin might have an effect on adhesion and incorporation of the tumor cells. Therefore, N-cadherin deficient SUM149-14-3 cell clones were generated by transduction with recombinant lentivirus, which expressed N-cadherin specific shRNA (Supplementary Figure S2). SUM149-scr-2-6 control cells, which contained an unspecific scrambled shRNA sequence were also generated. SUM149-sh14-3 showed a 97% downregulation of N-cadherin mRNA compared to the control cells, but also a 90% reduction of VE-cadherin mRNA (data not shown). In order to investigate the potential influence of N-cadherin on the interaction between tumor and endothelial cells, adhesion and incorporation experiments were carried out with SUM149-scr2-6 and N-cadherin deficient SUM149-14-3 cells. We observed a 42.5% reduced number of SUM149-sh14-3 cells that adhered to HUVEC as compared to control SUM149-2-6 cells (Supplementary Figure S2A). The results of the incorporation experiment revealed that SUM149-sh14-3 cells incorporate less frequently into HUVEC monolayers than SUM149-scr-2-6 cells. At 15 h after the beginning of the experiment, an average of 62% of the SUM149-sh14-3 cells and 82% of the SUM149-scr2-6 cells had incorporated in the HUVEC. The result of the two-way ANOVA test indicates that the time as well as the tumor cell lines (SUM149-sh14-3 or SUM149-scr2-6 cells) have an effect on incorporation (Supplementary Figure S2B). Thus, N-cadherin deficiency results in a reduction of both tumor cell adhesion and tumor cell incorporation into the HUVEC monolayer.

### 2.9. BT20, SUM149PT, SUM149-Scr-1 and SUM149-sh5-21 Cells Interact With Endothelial VE-Cadherin during Incorporation

Next, we investigated whether VE-cadherin is present at the site of tumor cell incorporation into the endothelial layer. Therefore, immunofluorescence analysis for VE-cadherin was performed for BT20, SUM149PT, SUM149-scr-1, SUM149-sh5-21 cells and the HUVEC, 21.5 h after the start of the incorporation experiment.

**Figure 4:**
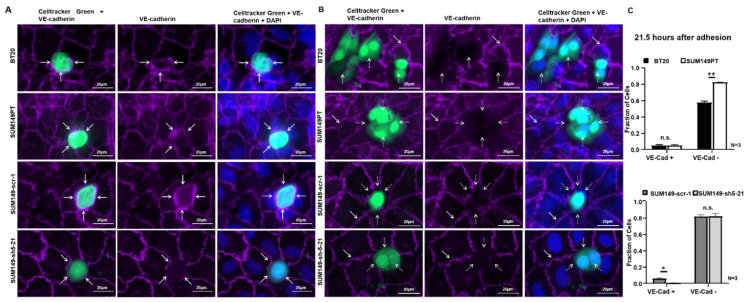
BT20, SUM149PT, SUM149-scr-1 and SUM149-sh5-21 cells destroy the VE-cadherin-mediated endothelial cell-cell contacts at a late stage of incorporation. The incorporation experiments were stained by immunofluorescence for VE-cadherin (purple) 21.5 h after the start of the experiment. Tumor cells green, 40x magnification and additional + 48% digital zoom. (**A**) VE-cadherin was present at the contacts between HUVEC and BT20, SUM149PT or SUM149-scr-1 cells, potentially reflecting homophilic interaction between tumor and endothelial cells (white arrows). At the contacts between HUVEC and SUM149-sh5-21 knockdown cells, only endothelial VE-cadherin was visible. Endothelial VE-cadherin between the HUVECs was also present in all images. We interpreted this as an early stage of incorporation; (**B**) BT20, SUM149PT, SUM149-scr-1 and SUM149-sh5-21 cells disrupted the VE-cadherin mediated HUVEC cell-cell contacts at the site of tumor cell incorporation. BT20, SUM149PT and SUM149-scr-1 cells did not express VE-cadherin on the surface (white dotted arrows); the VE-cadherin staining between adjacent HUVECs was however preserved (white arrows). This might be interpreted as the late stage of the incorporation process; (**C**) Quantification of incorporation by VE-cadherin immunofluorescence analysis 21.5 h after the start of the experiment. Fraction of tumor cells with endothelial VE-cadherin at junctions to HUVEC (VE-Cad +) and tumor cells that disrupted endothelial VE-cadherin (VE-Cad −). Bars represent standard deviation; n.s. not significant, * *p* ≤ 0.05; ** *p* ≤ 0.01; statistical analysis was conducted using two-way ANOVA.

After the tumor cells had integrated into the HUVEC monolayer, endothelial VE-cadherin was present at the contacts between tumor cells and endothelial cells. VE-cadherin was preserved between neighboring HUVEC, and endothelial VE-cadherin was detected at the contacts of HUVEC and BT20, SUM149PT and SUM149-scr 1 cells (Figure 4A). In some places, it appeared that endothelial VE-cadherin and tumor cell VE-cadherin interact, as judged from the location and strength of the fluorescence signal (Figure 4A, white arrows). In the case of the SUM149-sh5-21 knockdown cells, less VE-cadherin was observed at the sites of contact to the adjacent HUVEC. Because the SUM149-sh5-21 cells express VE-cadherin only weakly, VE-cadherin staining at the cell contacts most likely reflects endothelial VE-cadherin expression only. Thus, endothelial VE-cadherin was often preserved at the contacts between the VE-cadherin knockdown tumor cells and HUVEC.

### 2.10. BT20, SUM149PT, SUM149-Scr-1 and SUM149-sh5-21 Cells Disrupt the Endothelial VE-Cadherin Barrier during Incorporation

At other locations in the same immunofluorescence analyses, VE-cadherin staining had disappeared at the sites of contact between BT20, SUM149PT, SUM149-scr-1 and SUM149-sh5-21 cells and HUVEC (Figure 4B). In contrast, endothelial VE-cadherin staining was always preserved between neighboring HUVEC (Figure 4B, white arrows). At the sites where BT20, SUM149PT, and SUM149 scr-1 cells had incorporated in the endothelial layer, they appeared to have displaced VE-cadherin from the endothelial cell-cell contacts. No VE-cadherin was detected at the surface of the BT20, SUM149PT and SUM149-scr-1 tumor cells (Figure 4B, white dashed arrows). VE-cadherin deficient cell clones SUM149-sh5-21 also incorporated into the HUVEC endothelium and disrupted the VE-cadherin mediated endothelial cell-cell contacts. When incorporated, BT20, SUM149PT, SUM149-scr-1 and SUM149-sh5-21 cells disrupted the VE-cadherin-mediated endothelial cell contacts and created gaps in the endothelium (Figure 4B, white dashed arrows).

To further investigate this process, we quantified the fraction of tumor cells that either showed endothelial VE-cadherin at the cell-cell contacts, or had disrupted the homophilic VE-cadherin contacts between HUVEC endothelial cells. At 21.5 h after the beginning of the experiment, 57.3% of the BT20 and 82.5% of the SUM149PT cells had disrupted endothelial VE-cadherin (Figure 4C). Both cell lines showed similar low fractions of tumor cells with VE-cadherin present at the contacts to endothelial cells (4.7 vs. 5%). Together with the results obtained at 15 h after the start of the incorporation assay (Figure 3B), this suggests that not all tumor cells incorporated but rather a plateau was reached. This could also be observed for SUM149-scr-1 and VE-cadherin deficient SUM149-sh5-21 cells, which showed 81.8% and 82% cells that had disrupted the endothelial VE-cadherin barrier at 21.5 h after the beginning of the experiment (Figure 4C). This result further supports the hypothesis that VE-cadherin has no quantitative influence on the incorporation.

Incorporation of tumor cells was also monitored also at earlier time points. Three hours after the beginning of the experiment, BT20 cells appeared to disrupt the VE-cadherin positive contacts between endothelial cells less frequently than SUM149PT cells (21% vs. 31.7%; n.s.), but showed more often VE-cadherin at the tumor cell-endothelial cell contacts (42.9% vs. 22.7%). This might be caused by the pronounced VE-cadherin expression at the surface of BT20 cells. However, this difference was no longer observed at 9 h after the beginning of the experiment; at this time point, both BT20 and SUM149 cells showed a lower fraction of cells with endothelial VE-cadherin at contacts (11.7% vs. 7.3%). Yet, similar to the 21.5 h time point (see above), a higher fraction of SUM149PT cells had disrupted the endothelial VE-cadherin barrier as compared to BT20 cells (60.9% vs. 44.5%) (Supplementary Figure S3A). SUM149-scr-1 cells showed a higher fraction of cells with VE-cadherin at the contacts to HUVEC as compared to VE-cadherin deficient SUM149-sh5-21 cells (3 h after adhesion: 14.5% vs. 0.9%; 9 h after adhesion: 8.2% vs. 0.5%). At both time points, no difference in the fraction of endothelial VE-cadherin contact disrupting tumor cells was detectable (Supplementary Figure S3B).

Taken together, our observations show that endothelial VE-cadherin can either be preserved at the tumor cell-endothelial cell contacts, or may get lost. Based upon the analysis performed at different time points after the seeding of cancer cells, it appears that this reflects a temporal sequence of events, where tumor cells first make contact with endothelial VE-cadherin and then disrupt the endothelial barrier.

## 3. Discussion

The development and availability of targeted therapies have greatly improved the treatment success in many malignant diseases. Yet, metastasis remains an urgent problem of anti-tumor therapy [3,17,18]. It is therefore of utmost importance to characterize the processes that lead to the metastatic dissemination of cancer cells. Here, we have addressed the role of VE-cadherin in tumor cell-endothelial cell interactions. Such interactions are crucial for the process of extravasation of cancer cells at the site of metastasis formation. Our results show that aberrant expression of VE-cadherin in human breast cancer cells enhances their adhesion to endothelial cells which may favor metastasis formation. However, the subsequent transmigration of tumor cells was not directly influenced by VE-cadherin. Moreover, while integrating into the endothelial monolayer, VE-cadherin positive breast cancer cells showed a behavior indicative of a multistep process: first, tumor cells incorporate into the endothelial monolayer without affecting endothelial VE-cadherin localization at the tumor cell-endothelial cell contacts; then, VE-cadherin is displaced from these contacts, leading to the formation of gaps that allow the passage of tumor cells. Thus, tumor cells incorporate into the endothelium and disrupt the VE-cadherin mediated endothelial junctions. The results suggest that tumor cell VE-cadherin plays a role in the initial steps of extravasation, but not during transmigration.

Aberrant VE-cadherin expression has been reported for a few tumor types, notably melanoma and mammary carcinoma [12,13,19]. In melanoma, VE-cadherin has been implicated in vasculogenic mimicry, a phenomenon that describes the formation of avascular channels by tumor cells that express certain typical endothelial proteins [19]. Yet, the relevance of vasculogenic mimicry has been disputed [20]. Various studies have addressed the role of aberrant VE-cadherin expression in breast cancer [12,13,14,16]. In a mouse mammary carcinoma model, we had observed previously that VE-cadherin was induced in malignant tumor cells during the epithelial-to-mesenchymal transition (EMT), and enhanced tumor cell proliferation in vitro and tumor growth in vivo [12]. Analysis of 392 human breast cancer specimens revealed that VE-cadherin was aberrantly expressed in approximately 60% of human breast cancers analyzed in a subset of cancer cells, indicating a relevant function in this tumor type [16]. Consistent with a role in promoting cancer growth and progression, VE-cadherin was identified as a biomarker of metastatic breast cancer in the blood of human patients [15]. Moreover, VE-cadherin RGD motifs were shown to promote metastasis in experimental breast cancer; an effect which was associated with increased adhesion to the extracellular matrix, enhanced proliferation and invasion of MDA-MB-468 breast cancer cells in vitro [21].

However, modulation of cell dynamics by VE-cadherin might be a function of tumor cell differentiation: on the one hand, the forced overexpression of VE-cadherin in poorly differentiated MDA-MB231 breast cancer cells profoundly changed the cellular phenotype from a more fibroblastoid to a more epithelial phenotype, indicative of mesenchymal-to epithelial transition (MET) [16]; on the other hand, overexpression of VE-cadherin in the differentiated and less aggressive MCF-7 cell line hardly affected their phenotype. Based on these results, it appeared that forced expression of VE-cadherin might promote the formation of functional adherens junctions between tumor cells, similar as in endothelial cells [16]. Thus, VE-cadherin might act in breast cancer cells in a context-dependent manner.

In this study, we focused on the role of VE-cadherin in tumor cell-endothelial cell interactions. Rather than overexpressing VE-cadherin in breast cancer cells, we chose to analyze its functions in human breast cancer cells which express VE-cadherin endogenously. We identified two triple negative cell lines which expressed VE-cadherin mRNA and protein, BT20 and SUM149PT. BT20 cells showed a strong VE-cadherin expression which was confined to the cell surface, similar to its expression pattern in vascular endothelial cells [6]. Interestingly, BT20 cells expressed both VE- and E-cadherin, but only low levels of N-cadherin which has been implicated in tumor cell invasion and metastasis [10,22]. SUM149PT cells, in contrast, expressed VE-cadherin less abundantly, and in addition to cell surface expression, the protein was partially distributed diffusely over the cell, reminiscent of the cytoplasmic expression pattern often observed in breast cancer specimens [16]. SUM149PT showed, in addition to VE- and E-cadherin, also N-cadherin expression as well. N-cadherin plays a central role in the development of an invasive and mesenchymal cancer cell phenotype [10,23], and has been shown to promote metastatic behavior of tumor cells [10,21,24,25]. Moreover, forced N-cadherin expression in low-invasive and E-cadherin expressing human MCF-7 breast cancer cells led to a strongly increased invasion and motility [10]. The observation that E-cadherin and N-cadherin can be co-expressed in aggressive carcinoma cells is consistent with the observation that E-cadherin does not necessarily have to get lost during EMT [26]. The coexpression of VE-cadherin and N-cadherin, or E- and N-cadherin has also been observed previously in aggressive murine Ras-transformed mammary carcinoma cells [12]. These observations show that the N-cadherin-mediated promotion of invasion and motility can be more dominant than the tumor suppressor effect of E-cadherin, and this may also apply to SUM149PT cells.

It was observed previously in murine mammary carcinoma cancer cells that VE-cadherin was localized at the cell-cell contacts, whereas N-cadherin was diffusely distributed over the cell. Knockdown of VE-cadherin resulted in the increased accumulation of N-cadherin at the intercellular junctions [13], suggesting that VE-cadherin could displace N-cadherin from cell junctions. However, the down-regulation of VE-cadherin in SUM149PT cells in the present study did not result in an increased accumulation of N-cadherin at the cell-cell contacts, indicating that the interaction of cadherins in tumor cells is complex. In contrast to its diffuse distribution in SUM149PT cells, VE-cadherin expression in BT20 cells was confined to the cell-cell contacts. It is tempting to speculate that this localization favors the adhesion of BT20 cells to HUVEC.

To investigate the potential functions in tumor cell endothelial cell interaction, we first compared tumor cell adhesion of BT20 and SUM149PT cells, which differ in their VE-cadherin expression levels and localization, to HUVEC endothelial cells. BT20 cells consistently adhered more frequently to HUVEC than SUM149PT cells (Figure 2A)). This result supports the hypothesis that VE-cadherin on tumor cells might promote their adhesion to endothelial cells which express VE-cadherin abundantly. The preferential localization of VE-cadherin at the cell surface of BT20 cells, as opposed to the more diffuse expression in SUM149PT cells, might enhance to this effect. It was reported previously that overexpression of N-cadherin in human MCF7 breast cancer cells promotes adhesion to HUVEC [10]. Thus, BT20 cells adhere better to HUVEC than SUM149PT cells despite low N-cadherin expression, but a significantly higher expression of VE-cadherin. It might therefore be speculated that the adhesion-promoting effect of VE-cadherin was particularly pronounced in BT20 cells.

Proof for the adhesion-promoting effect of VE-cadherin comes from our experiments in which VE-cadherin expression was silenced in human breast cancer cell lines. Unfortunately, it was not possible to generate stable VE-cadherin deficient BT20 cells, possibly because BT20 cells do not tolerate VE-cadherin deficiency. In contrast, we were able to generate VE-cadherin knockdown SUM149PT cell clones (Figure 1A). These showed 41% or 42% reduced adhesion as compared to SUM149PT scramble control clones, respectively (Figure 1B and Figure 2B,C). Furthermore, we observed a 31.2% reduced adhesion of the independently generated SUM149-sh5 VE-cadherin knockdown cell line as compared to control cells (Figure 2D). This demonstrates that aberrant VE-cadherin expression enhances tumor cell adhesion to HUVEC.

We hypothesized that VE-cadherin might promote interaction between tumor and endothelial cells through the formation of homophilic interactions between endothelial and tumor cell VE-cadherin (Figure 4A). To address this question, we performed time lapse microscopy of BT20, SUM149PT, SUM149-scr-1, SUM149-sh5-21 and SUM149-sh5-17 cells on a HUVEC monolayer and monitored the process of tumor cell incorporation into the endothelial monolayer. We observed that tumor cells of these lines were incorporated into the HUVEC endothelium. Yet, a broad and non-cell line specific spectrum of incorporation behavior could be observed (Figure 3A,D,E). Interestingly, SUM149PT cells incorporated more efficiently into the HUVEC monolayer than BT20 cells, despite a lower frequency of adhesion. At 15 h after the start of the experiment, an average of approximately 61% of the BT20 cells and 83% of the SUM149PT cells had been incorporated in the HUVEC. This suggests that tumor cell incorporation is not simply a function of efficient adhesion, but also depends also on different parameters. It is tempting to speculate that the presence of N-cadherin on human breast cancer cells, which is associated with increased motility, migration, invasion and metastasis [10,23], could make a difference. BT20 cells showed a lower invasive potential and motility compared to N-cadherin expressing human breast cancer cells [23]. Furthermore, it has been observed in melanoma cells that the downregulation of N-cadherin results in a reduction of transendothelial migration [27]. Thus, N-cadherin apparently not only influences the motility, migration and invasion of human breast cancer cells, but also the incorporation into the endothelium. A potential reason for the significantly lower incorporation of BT20 cells compared to SUM149PT cells might be the lack of N-cadherin expression in BT20 cells. This hypothesis is supported by our results obtained with N-cadherin deficient SUM149PT cells, which revealed that that the loss of N-cadherin reduces adhesion as well as the incorporation (Supplementary Figure S2A,B). Because the knockdown of N-cadherin also resulted also in a down-regulation of VE-cadherin, it is unclear to which extent N-cadherin and VE-cadherin contributed to the adhesion to the endothelium.

When SUM149PT control cells were compared to the VE-cadherin deficient SUM149PT cell clones, there was no difference in the proportion of incorporated cells (Figure 3B,C). Therefore, it can be concluded that VE-cadherin has no quantitative influence on the incorporation of SUM149PT cells. Consistent with this conclusion, a promoting effect of RGD peptide sequences of VE-cadherin in human MDA-MB-468 breast cancer cells on proliferation, adhesion to collagen 1, invasion and metastasis could be shown; however, transendothelial migration was not affected [21], consistent with our own observations.

Furthermore, we could show by immunofluorescence analysis that the VE-cadherin of the breast cancer cell lines tested can be associated with endothelial VE-cadherin during the incorporation process (Figure 4A). At the contacts between HUVEC and tumor cells, endothelial VE-cadherin was preserved. Our observation that VE-cadherin can be present at the contacts between tumor cells and endothelial cells would be consistent with localization in homophilic cell contacts. It should be noted, however, that it is was not possible to clearly distinguish endothelial cadherin and tumor cell cadherin in this assay.

Our observations contrast with a report by Hamilla et al. who described a VE-cadherin independent incorporation of human MDA-MB-231 breast cancer cells; no endothelial VE-cadherin was observed at the contacts between HUVEC and incorporated MDA-MB-231 breast cancer cells, where the incorporation was associated with the dislocation of the VE-cadherin mediated cell-cell contacts between the endothelial cells [28]. The fact that these authors did not observe VE-cadherin expression at the tumor cell endothelial cell contacts might be explained by the different cell lines used: our experiments were designed to specifically address the behavior of VE-cadherin positive breast cancer cells. It is reasonable to assume that tumor cells that express VE-cadherin, like the BT20 and SUM149PT cells used in our study, show a somewhat different incorporation behavior as compared to VE-cadherin negative tumor cell lines. However, we also observed an absence of endothelial VE-cadherin at the sites of endothelial cell-tumor cell contact. The incorporation of BT20, SUM149PT, SUM149-scr-1 and SUM149 sh5-21 cells generated gaps in the endothelial cell layer which might serve as a simple passageway for transendothelial migration of following tumor cells (Figure 4B), most likely though paracellular pathways [28,29]. It has been described that triple-negative MDA-MB-231 breast cancer cells undergo paracellular transmigration after endothelial cell-cell adhesion disengaged at spots of tumor cell intravasation [17]. Paracellular transmigration is the most common mechanism of tumor cells in vitro and requires the dislocation of endothelial cell-cell-contacts [9].

**Figure 5:**
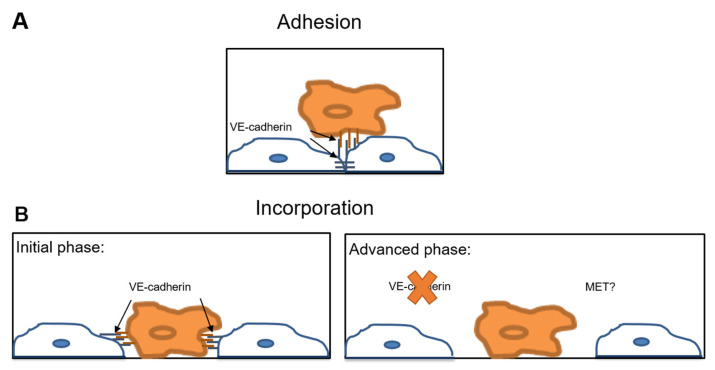
VE-cadherin interactions at the tumor cell-endothelial cell interface. Hypothetical model illustrating the VE-cadherin mediated interactions between tumor cells and endothelial cells during extravasation. (**A**) The adhesion of the tumor cell (orange) to the endothelial cell (blue) is mediated by the homophilic interaction between VE-cadherin on tumor cells and endothelial VE-cadherin. VE-cadherin facilitates tumor cell adhesion to the endothelium; (**B**) Initial incorporation phase: adherent tumor cells (orange) incorporate into the endothelium (blue). VE-cadherin might be involved in this initial phase of incorporation after mediating the contact between tumor and endothelial cells. Advanced phase: In the course of incorporation, endothelial cells (blue) downregulate VE-cadherin at the sites of contacts to the tumor cells (orange). The disruption of the VE-cadherin mediated endothelial cell contacts creates a gap in the endothelial layer through which tumor cells can transmigrate paracellularly. MET, mesenchymal-to-epithelial transition at the site of metastasis.

These seemingly contrasting observations might be reconciled if one assumes that transendothelial migration of tumor cells is a multistage process. Assuming that tumor cells make contact with and adhere to endothelial cells via VE-cadherin, these contacts might be preserved during the initial phase of tumor cell incorporation. In the course of this process, VE-cadherin at the cell-cell contacts would disappear. Later on, tumor cells would disrupt the endothelial layer and generate gaps for the passage of tumor cells (Figure 5). 

In conclusion, we identified VE-cadherin as an important effector of transendothelial migration and potentially of metastasis. Aberrant VE-cadherin expression in breast cancer cells can promote their adhesion to endothelial cells at least in the initial phase of incorporation into the endothelial monolayer. This interaction might be targeted in triple negative breast cancer by VE-cadherin specific antibodies. The results of our work support the conclusion that aberrant VE-cadherin expression in breast cancer promotes tumor-endothelial interactions and identify it as potential target for anti-tumor therapy of metastatic breast cancer.

## 4. Materials and Methods

### 4.1. Cell Culture

Triple negative breast cancer cell lines, BT20 (CLS Cell Line Service, Eppelheim, Germany) and SUM149PT (Asterand, Royston, UK), and the derivatives SUM149-scr-1, SUM149-sh5-21, and SUM149-sh5-17 cells, were cultured in Dulbecco´s Modified Eagle Medium (DMEM; Gibco Life Technologies, Grand Island, NY, USA) supplemented with 10% FCS. Human umbilical vein cells (HUVEC) were grown in Clonetics Endothelial Growth Medium (EGM; Promocell, Heidelberg, Germany). The medium was changed every 2 days. When cells reached confluency, they were split in a 1:3 ratio. The collection of primary human umbilical vein endothelial cells (HUVEC) was approved by the ethical review board of the Medical Faculty Carl Gustav Carus of the TU Dresden (EK124082003). Primary cultures of HUVEC were isolated using 0.5% collagenase II solution (Worthington Biochemical Corp. Lakewood, NJ, USA) [30].

### 4.2. Generation of VE-Cadherin Knockdown Breast Cancer Cells

Lentiviral vectors containing human VE-cadherin shRNA were generated essentially as described in [12,13] and used to transduce SUM149PT cells (Supplementary Table S6). A lentivirus containing an unspecific scramble sequence was used as a control. After transduction, cells were FACS sorted and seeded at low density in 96-well plates to generate single cell clones. The resulting single cell clones were analyzed by RT-PCR and qRT-PCR for expression of VE-cadherin, as described below. Clones SUM149-sh5-21 and SUM149-sh5-17 showed the strongest reduction in VE-cadherin expression as compared to SUM149-scr-1 scramble control cells (Figure 1A,B) and were used for further analysis.

In addition, to exclude potential positional effects resulting from of lentivirus integration, a VE-cadherin deficient SUM149-sh5 cell line was generated by lentiviral transduction and propagation of a pool of SUM149PT cells that expressed VE-cadherin specific shRNA (Supplementary Table S6). This cell line represents a mixture of different individually transduced SUM149PT cells. Cells were analyzed by qRT-PCR and qRT-PCR for expression of VE, N- and E-cadherin. The efficiency of VE-cadherin downregulation was determined compared to SUM149 ctrl control cells (Figure 1C,D).

### 4.3. RNA Isolation and Reverse Transcription-Polymerase Chain Reaction (RT-PCR) Analysis

Cells were grown to confluency in 6-well-plates or 6 cm Petri dishes. Total RNA was isolated from cell lysates using GeneMatrix Universal RNA Purification Kit (Roboklon GmbH, Berlin, Germany) according to the instructions of the manufacturer. Aliquots of 1000 ng total RNA were reverse transcribed using Superscript II enzyme (Invitrogen, Carlsbad, CA, USA). RT-PCR for human VE-, N- and E-cadherin was performed in a C1000 Touch Thermal Cycler (Bio-Rad Laboratories GmbH, Munich, Germany) using 50 ng cDNA. PCR primers, reagents and the PCR protocol used are described in Supplementary Table S7–S9.

### 4.4. Quantitative Reverse Transcription-PCR (qRT-PCR)

qRT-PCR was performed essentially as described in [13] according to the manufacturer’s instructions (Bio-Rad Laboratories GmbH, Munich, Germany). Using 1 µL cDNA generated from reverse transcription of 10 ng RNA with 5 µL SYBR Green Supermix (Sso7d fusion polymerase, dNTPs, MgCl_2_, SYBR Green I dye, enhancer, stabilizer and passive reference dyes), 2.8 µL HPLC H_2_O and 1.2 µL of the respective primers (Supplementary Table S10) in a total volume of 10 µL. Each sample was determined in triplicate. Reference genes used were TATA box binding protein (TBP), elongation factor-2 (EF2) and hypoxanthine phosphoribosyl transferase (HPRT). The PCR program is shown in Supplementary Table S11. For the evaluation, the ΔΔCt-method was used [31].

### 4.5. Immunofluorescence

For the immunofluorescence analysis, the cells were grown to confluency on 6- or 12-chamber-slides (Ibidi GmbH, Martinsried, Germany). Cells were then fixed in 500 µL 4% formalin and stained overnight with the following primary antibodies: VE-cadherin F8 mouse monoclonal IgG (Santa Cruz Biotechnology, Heidelberg, Germany), purified mouse anti-N-cadherin (BD Transduction Laboratories, Heidelberg, Germany) and rabbit anti E-cadherin antibody EP700Y (NOVUS Biologicals, Wiesbaden Nordenstadt, Germany). All primary antibodies were used in 1:100 dilution. Secondary antibodies used were Alexa Fluor 568 dye anti-mouse for VE-cadherin, Alexa Fluor 568 dye anti-mouse for N-cadherin, Alexa Flour 568 dye anti-rabbit for E-cadherin, and Alexa Fluor 647 dye anti-mouse for E-cadherin. All secondary antibodies were from ThermoFisher Scientific, Dreieich, Germany, and used in a 1:200 dilution. The incubation time for secondary antibodies was one hour. Cell nuclei were stained with 4.6-diamidino-2-phenylindole (DAPI, Thermo Scientific GmbH, Darmstadt Deutschland) in 1:500 dilution. Images were taken with a Zeiss Observer Z.1 ApoTome I microscope (Carl Zeiss Microscopy, Jena, Germany). All primary and secondary antibodies are shown in Supplementary Table S12. For the quantitative immunofluorescence analysis, the percent area positive for VE-, N- and E-cadherin was determined using ImageJ/Fiji software (Wayne Rasband, Bethesda, MD, USA).

### 4.6. Adhesion Assay

Tumor cells were grown to confluency in 24-well-plates and stained with 250 µL of Celltracker Orange (ThermoFisher Scientific, Dreieich, Germany), diluted 1:1000 in DMEM (+10% FCS) for 60 min at 37 °C and 5% CO_2_. Then, tumor cells were trypsinized, and aliquots of 100,000 cells were added to a confluent HUVEC monolayers in one well of the 6-well-plate. The incubation of the tumor cells took place for 30 min. To remove unbound tumor cells, plates were washed four times with PBS. Cells were fixed with 1 mL of 4% formalin. Subsequently, defined areas (63.5 mm^2^) were recorded using a Zeiss Axiovert ApoTome II microscope (Carl Zeiss Microscopy, Jena, Germany), and the number of adherent cells was determined using ImageJ/Fiji software (Wayne Rasband, Bethesda, MD, USA).

An alternative method for the quantification of tumor cell adhesion was performed using a fluorescence intensity-based approach. Tumor cells were cultured, stained, and detached as described above. Then, 2500 tumor cells were added to a confluent HUVEC monolayer per well of a 96-well-plate. The cells were incubated for 1 h. Before and after the respective washing steps with PBS, the fluorescence intensities were measured for each well using the CLARIOstar microplate reader (BMG Labtech, Ortenberg, Germany). Fluorescence intensity was shown to correlate to tumor cell numbers. The two-time measurement of fluorescence intensity allowed the calculation of tumor cell adhesion as a percentage of adhering cells from all tumor cells in suspension.

### 4.7. Incorporation Assay

Tumor cells were seeded on 24-well-plates and HUVEC on 3 chamber slides. After reaching confluency, the cells were fluorescently labelled with 250 µL Celltracker Green for tumor cells and Celltracker Orange (both from ThermoFisher Scientific, Dreieich, Germany) for HUVEC in 1:1000 dilution DMEM and EGM for 60 min at 37 °C and 5% CO_2_. Aliquots of tumor cells (50,000) were added to the confluent monolayer of HUVEC and incubated for 30 min at 37 °C and 5% CO_2_. To remove unbound tumor cells, the medium was removed and replaced by fresh EGM. The recording started 1 h after the addition of the tumor cells to the HUVEC monolayer. Interactions between tumor cells and HUVEC were monitored on an average area of 2.75 mm^2^ to 2.94 mm^2^ on the Zeiss Observer Z.1 live cell-imaging microscope (Carl Zeiss Microscopy, Jena, Germany) every 10 min for the next 15 h. 21.5 h later, the assay was fixed with 1 mL of 4% formalin. Then, immunofluorescence staining for VE-cadherin was performed as described above. In addition, incorporation assays were performed for different time points at 3 and 9 h after the start of the adhesion experiment. The analyses of the live cell images were done by visual inspection and ImageJ/Fiji (Wayne Rasband, Bethesda, MD, USA). Imaging experiments were performed with the support of the Core Facility Cellular Imaging (CFCI) at the Faculty of Medicine Carl Gustav Carus Dresden.

## Data Availability

Data is contained within the article or supplementary material.

## Supplementary Materials

The following are available online at https://www.mdpi.com/article/10.3390/ijms22116049/s1.
