# Supplementary material for: The Influence of VE-Cadherin on Adhesion and Incorporation of Breast Cancer Cells into Vascular Endothelium
Source: Int J Mol Sci. 2021 Jun 3;22(11):6049. doi: 10.3390/ijms22116049 (PMC8199973; doi:10.3390/ijms22116049)
Supplement: Supplementary file 1 [file ijms-22-06049-s001.zip › ijms-1210206-supplementary.pdf]

Supplementary Information

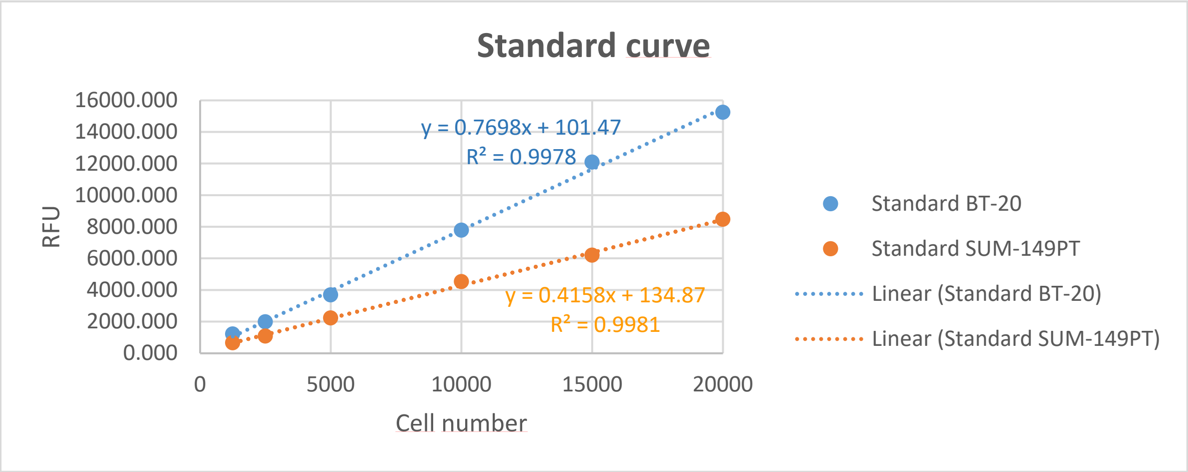

Figure S1: Fluorescence intensity standard curve
Fluorescence intensity is proportional to tumor cell number with r2>0.99 for both BT-20 (blue) and SUM149PT cells (orange). Fluorescence intensity [RFU] as a function of tumor cell number.

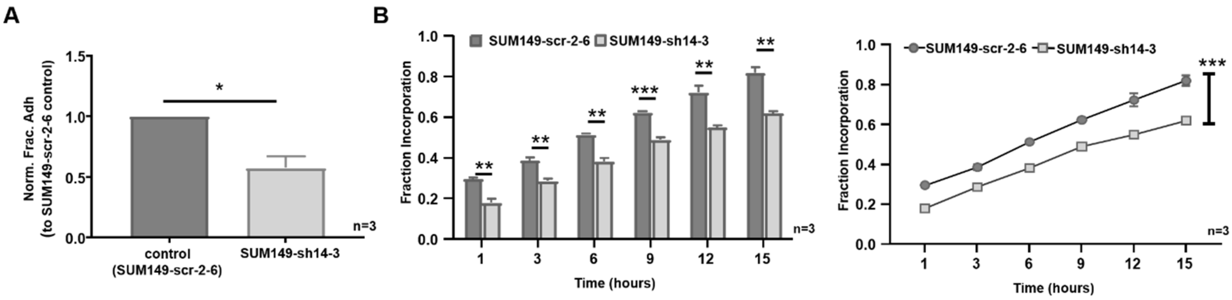

Figure S2. The adhesion and incorporation of SUM149PT cells into HUVEC is N-cadherin dependent

N-cadherin deficient SUM149-14-3 cell clones were generated by transduction with recombinant lentivirus, which expressed N-cadherin specific shRNA (Supplementary Table 6). SUM149-scr-1 control cells, which contained an unspecific scrambled shRNA sequence were also generated. SUM149-sh14-3 showed a 97% downregulation of N-cadherin mRNA compared to the control cells. The SUM149-sh14-3 cells also had a 90% reduced of VE-cadherin mRNA expression relative to SUM149-scr-2-6 cells (data not shown). **(A)** Identical numbers of tumor cell (100,000) were fluorescently labelled and incubated on a monolayer of HUVEC for 30 minutes. Normalized Fraction Adhesion (Norm. Frac. Adh.) to control. There was a 42.5 % reduced number of cells that had adhered to HUVEC for SUM149-sh14-3 to SUM149-2-6 cells. **(B)** 50,000 tumor cells were fluorescently labeled and placed on a monolayer of HUVEC. The incorporation was recorded on an area of 2.94 mm^2 over 15 hours with microscope focus on the HUVEC monolayer. Fraction of Incorporation at 1, 3, 6, 9, 12 and 15 hours after the start of adhesion experiment. SUM149-scr-2-6 cells incorporated more often into the HUVEC monolayer as compared to N-cadherin deficient SUM149-sh14-3 cells. The incorporation of these two cell lines increased over time. Bars represent standard deviation; (n.s. not significant, *p<0.5, **p≤0.01, ***p≤0.001); statistical analysis by unpaired welch-t-test and two-way ANOVA.

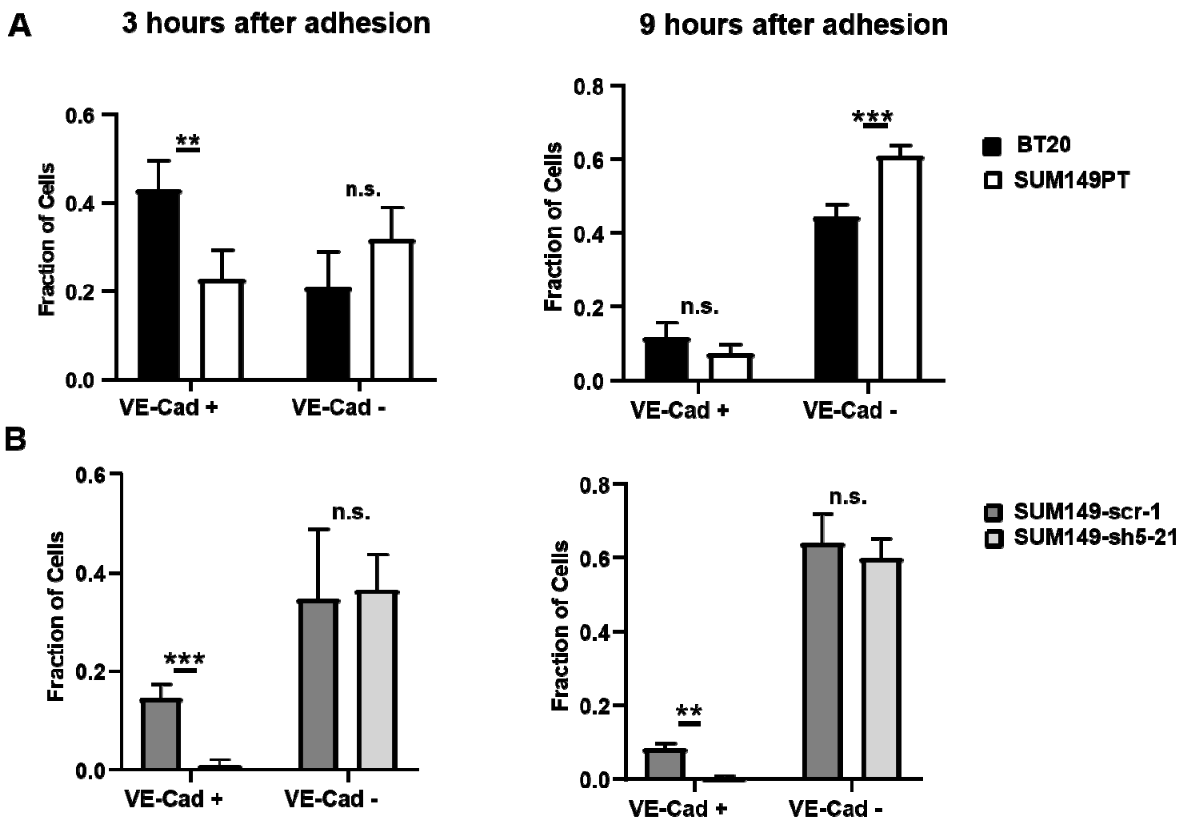

Figure S3. VE-cadherin interaction of HUVEC and tumor cells at different time points of incorporation

Quantification of incorporation, as analyzed by VE-cadherin immunofluorescence analysis at 3 and 9 hours after the start of the incorporation experiment. Fraction of tumor cells with VE-cadherin at junctions to HUVEC (VE-Cad +), or tumor cells that had disrupted endothelial VE-cadherin positive cell contacts (VE-Cad -). For calculation, 4 different spots were analyzed. **(A)** At 3 hours after the beginning of the adhesion experiment, BT20 and SUM149PT cells differed in the Fraction of cells that had VE-cadherin at the sites of contact to HUVEC (42.9 % vs 22.2 %), but not in the fraction of cells that had disrupted endothelial VE-cadherin (21 % vs. 31.7 %; n.s.). At 9 hours, BT20 cells and SUM149PT cells showed similar fractions of cells with VE-cadherin at junctions to HUVEC (11.7% vs 7.3%). Yet, a higher fraction of SUM149PT cells had disrupted endothelial VE-cadherin (60.9 % vs. 44.5 %). **(B)** Three hours after the beginning of the adhesion experiment, SUM149-scr-1 control cells showed more often endothelial VE-cadherin staining at the sites of contact to HUVEC as compared to VE-cadherin deficient SUM149-sh5-21 cells (14.5 % vs 0.09 %). At 9 hours, this effect persisted (8.2 % vs 0.05 %). At both time points, no difference in the fraction of cells that had disrupted endothelial VE-cadherin was observed. Bars represent standard deviation; n.s. not significant, *p≤0.05; **p≤0.01; ***p≤0.001; statistical analysis by two-way Anova.

Table S1. Fraction Adhesion BT20 and SUM149PT

	adherent BT20 cells	Ratio adherent BT20 cells to average adherent BT20 cells	normalized Fraction Adhesion BT20 cells	adherent SUM149PT cells	Ratio adherent SUM149PT cells to average adherent SUM149PT cells cells	normalized Fraction Adhesion SUM149PT cells
**Exp. 1**	594<br>471<br>562	1,09 (594/542,5)<br>0,87 (472/542,5)<br>1,04 (562/542,5)	1,00	161<br>339<br>252	0,30 (161/542,5)<br>0,62 (339/542,5)<br>0,47 (252/542,5)	0,46
**Exp. 2**	235<br>241<br>254	0,97 (235/243,)<br>0,99 (241/243)<br>1,04 (254/243)	1,00	54<br>40<br>217	0,224 (54/243,)<br>0,166 (40/243)<br>0,89 (217/243)	0,43
**Exp 3**	824<br>981<br>789	0,95 (824/864,5)<br>1,13 (981/864,5)<br>0,91 (789/864,5)	1,00	686<br>711<br>637	0,79 (686/864,5)<br>0,82 (711/864,5)<br>0,74 (637/864,5)	0,78
**Exp 4**	237<br>212<br>183	1,12 (237/201,5)<br>1,01 (212/201,5)<br>0,87(183/201,5)	1,00	51<br>40<br>42	0,24 (51/201,5)<br>0,19(40/201,5)<br>0,20 (42/201,5	0,21

Table S2 Fraction Adhesion SUM149-scr-1 and SUM149-sh5-21

	adherent SUM149-scr-1 cells	Ratio adherent SUM149-scr-1 cells to average adherent SUM149-scr-1 cells	normalized Fraction Adhesion SUM149-scr-1 cells	adherent SUM149-sh5-21 cells	Ratio adherent SUM149-sh5-21 to average adherent SUM149-scr-1 cells	normalized Fraction Adhesion SUM149-sh5-21 cells
**Exp. 1**	632<br>771<br>594	0,95 (632/665,6)<br>1,16 (771/665,6)<br>0,89 (594/665,6)	1,00	556<br>541<br>462	0,84 (556/665,6)<br>0,81 (541/665,6)<br>0,69 (462/665,6)	0,78
**Exp. 2**	185<br>195<br>134	1,08 (185/171,47)<br>1,14 (195/171,47)<br>0,78 (134/171,47)	1,00	85<br>56<br>102	0,49 (85/171,47)<br>0,33 (56/171,47)<br>0,59 (102/171,47)	0,47
**Exp 3**	268<br>88<br>101	1,76 (268/152,2)<br>0,58 (88/152,2)<br>0,66 (101/152,2)	1,00	45<br>42<br>61	0,30 (45/152,2)<br>0,28 (42/152,2)<br>0,40 (61/152,2)	0,32
**Exp 4**	306<br>334<br>360	0,91 (306/333)<br>1,00 (334/333)<br>1,08 (360/333)	1,00	263<br>217<br>263	0,79 (263/333)<br>0,65 (217/333)<br>0,79 (263/333)	0,74

Table S3 Fraction Adhesion SUM149-scr-1 and SUM149-sh5-17

	adherent SUM149-scr-1 cells	Ratio adherent SUM149-scr-1 cells to average adherent SUM149-scr-1 cells	normalized Fraction Adhesion SUM149-scr-1 cells	adherent SUM149-sh5-17 cells	Ratio adherent SUM149-sh5-17 to average adherent SUM149-scr-1 cells	normalized Fraction Adhesion SUM149-sh5-17 cells
**Exp. 1**	312<br>178<br>225	1,31 (312/238)<br>0,75 (178/238)<br>0,94 (225/238)	1,00	178<br>168<br>191	0,75 (178/238)<br>0,71 (168/238)<br>0,80 (191/238)	0,75
**Exp. 2**	272<br>145<br>262	1,19 (272/227,9)<br>0,66 (145/227,9)<br>1,15 (262/227,9)	1,00	80<br>85<br>86	0,35 (80/227,9)<br>0,37 (85/227,9)<br>0,38 (86/227,9)	0,37
**Exp. 3**	59<br>63<br>72	0,92 (59/64,8)<br>0,98 (63/64,8)<br>1,10 (72/64,8)	1,00	32<br>32<br>28	0,49 (32/64,8)<br>0,50 (32,/64,8)<br>0,43 (28/64,8)	0,47
**Exp. 4**	103<br>123<br>91	(109/107,53)<br>(123/107,53)<br>(91/107,53)	1,00	69<br>99<br>83	0,64 (69/107,53)<br>0,92 (99/107,53)<br>0,77 (83/107,53)	0,78

Table S4: Fraction Incorporation BT20 and SUM149PT

	BT20: (incorporated BT 20 cells /total cells)	Average Fraction Incorporation of BT20 cells from n_1 to n_3 (area: 2,75 mm^2)	SUM149PT: (incorporated SUM149PT cells / total cells)	Average Fraction Incorporation of SUM149PT cells from n_1 to n_3 (area: 2,75 mm^2)	Welch-t-test
**1 h**	n_1=0.15 (27/176)<br>n_2=0.10 (18/177)<br>n_3=0.12 (34/285)	0.123	n_1=0.17 (20/120)<br>n_2=0.14 (16/113)<br>n_3=0.19 (27/145)	0.167	p=0.1026
**3 h**	n_1=0.26 (47/180)<br>n_2=0.22 (29/133)<br>n_3=0.23 (63/276)	0.236	n_1=0.26 (50/189)<br>n_2=0.35 (36/102)<br>n_3=0.34 (62/185)	0.311	p=0.0907
**6 h**	n_1=0.38 (66/174)<br>n_2=0.36 (44/121)<br>n_3=0.34 (96/281)	0.358	n_1=0.44 (87/200)<br>n_2=0.51 (31/61)<br>n_3=0.54 (100/185)	0.489	p=0.0309
**9 h**	n_1=0.47 (88/188)<br>n_2=0.46 (59/128)<br>n_3=0.44 (133/301)	0.454	n_1=0.56 (121/218)<br>n_2=0.64 (29/45)<br>n_3=0.69 (174/254)	0.627	p=0.0386
**12 h**	n_1=0.53 (102/192)<br>n_2=0.55 (82/150)<br>n_3=0.55 (188/344)	0.542	n_1=0.71 (152/214)<br>n_2=0.73 (30/41)<br>n_3=0.74 (213/286)	0.730	p=0.0001
**15 h**	n_1=0.58 (111/192)<br>n_2=0.63 (97/154)<br>n_3=0.61 (252/414)	0.605	n_1=0.88 (207/235)<br>n_2=0.82 (37/45)<br>n_3=0.80 (226/283)	0.835	p=0.0028

Table S5: Fraction Incorporation SUM149-scr-1 and SUM149-sh5-21

	SUM149-scr-1 (incorporated SUM149-scr-1 cells /total cells)	Average Fraction Incorporation of SUM149-scr-1 cells from n_1 to n_3 (area: 2,94 mm^2)	SUM149-sh5-21 (incorporated SUM149-sh5-21 cells /total cells)	Average Fraction Incorporation of SUM149-sh5-21 cells from n_1 to n_3 (area: 2,94 mm^2)	Welch-t-test
**1 h**	n_1=0.17 (37/213)<br>n_2=0.22 (50/231)<br>n_3=0.19 (36/187)	0.195	n_1=0.15 (22/143)<br>n_2=0.23 (57/249)<br>n_3=0.16 (26/163)	0.189	p= 0.6757
**3 h**	n_1=0.31 (65/208)<br>n_2=0.36 (81/228)<br>n_3=0.31(70/227)	0.326	n_1=0.33 (41/126)<br>n_2=0.33 (83/249)<br>n_3=0.32 (53/164)	0.328	p= >0.9999
**6 h**	n_1=0.51 (100/196)<br>n_2=0.48 (110/230)<br>n_3=0.46 (99/216)	0.481	n_1=0.51 (66/129)<br>n_2=0.46 (110/238)<br>n_3=0.46 (72/156)	0.474	p= 0.7783
**9 h**	n_1=0.66 (120/182)<br>n_2=0.65 (142/217)<br>n_3=0.62 (137/222)	0.643	n_1=0.61 (82/134)<br>n_2=0.62 (146/234)<br>n_3=0.61 (90/147)	0.617	p= 0.1212
**12 h**	n_1=0.72 (119/165)<br>n_2=0.74 (152/205)<br>n_3=0.75 (152/203)	0.738	n_1=0.69 (82/118)<br>n_2=0.73 (162/223)<br>n_3=0.68 (95/140)	0.7	p= 0.1235
**15 h**	n_1=0.79 (118/149)<br>n_2=0.82 (186/228)<br>n_3=0.84 (173/206)	0.818	n_1=0.79 (77/97)<br>n_2=0.81 (169/209)<br>n_3=0.80 (112/140)	0.8	p= 0.3748

Table S6: shRNA Sequences for VE- and N-cadherin

Gene	shRNA Sequence
VE-cadherin shRNA	Sense:<br>5‘-CGCGTCCCAGACATCAATGACAACTTCTTCAAGAGAGAAGTTGTCATTGATGTCTTTTTTGGAAAT-3’<br>Anti-Sense:<br>3’-AGGGGTCTGTAGTTACTGTTGAAGAAGTTCTCTCTTCAACAGTAACTACAGAAAAAACCTTTAGC-5’
VE-cadherin Scramble	Sense:<br>5‘-CGCGTCCCAGGATCTCGACTTATCATGTTCAAGAGACATGATAAGTCGAGATCCTTTTTTGGAAAT-3’<br>Anti-Sense:<br>3’-AGGGGTCCTAGAGCTGAATAGTACAAGTTCTCTGTACTATTCAGCTCTAGGAAAAAACCTTTAGC-5’
N-cadherin shRNA	Sense:<br>5‘-CGCGTCCCAGTGCAACAGTATACGTTATTCAAGAGATAACGTATACTGTTGCACTTTTTTGGAAAT-3’<br>Anti-Sense:<br>3’-AGGGGTCACGTTGTCATATGCAATAAGTTCTCTATTGCATATGACAACGTGAAAAAACCTTTAGC-5’
N-cadherin Scramble	Sense:<br>5‘CGCGTCCCACCGCTAGTCCGTCTATTGTTCAAGAGACAATAGACGGACTAGCGGTTTTTTGGAAAT-3’<br>Anti-Sense:<br>3’-AGGGGTGGCGATCAGGCAGATAACAAGTTCTCTGTTATCTGCCTGATCGCCAAAAAACCTTTAGC-5’

Table S7. Master Mix for RT-PCR

Substance	Volume for 1x in µL
H_2O	13.04
RT-PCR buffer Promega green	4.0
dNTP Mix 10 mM	0.4
$MgCl_2$	1.2
Primer forward	0.13
Primer reverse	0.13
Taq DNA Polymerase	0.1

Table S8. RT-PCR Primers

Primer	Sequence
h-gapdh-fw 983 bp<br>h-gapdh-rev	TGAAGGTCGGAGTCAACGGATTGGGT<br>CATGTGGGCCATGAGGTCCACCAC
h-ve-cad-fw 430 bp<br>h-ve-cad-rev	GGCCTGTGTTCACGCATCGGTTGT<br>GTTCTGGGGCTCATCTGGGTCCTC
h-n-cad-fw 416 bp<br>h-n-cad-rev	CACTGCTCAGGACCCAGAT<br>TAAGCCGAGTGATGGTCC
h-e-cad-fw 102 bp<br>h-e-cad-rev	TCCACAGCCACCGTCACCGT<br>GCCCACGCCAAAGTCCTCGG
h-beta-actin fw 309 bp<br>h-beta-actin rev	GCCAACCGCGAGAAGATGACCC<br>CGAAGTCCAGGGCGACGTAGC

Table S9. RT-PCR programs

	Step	Temperature	Time	Number Cycles
GAPDH	1<br>2<br>3<br>4<br>5<br>6	94<br>94<br>68<br>72<br>72<br>4	2 min<br>30 s<br>30 s<br>1 min<br>10 min<br>-	Steps 2-4 repeated 25 times
VE-cadherin	1<br>2<br>3<br>4<br>5<br>6	94<br>94<br>62<br>72<br>72<br>4	2 min<br>15 s<br>20 s<br>10 s<br>10 min<br>-	Steps 2-4 repeated 30-35 times
N-cadherin	1<br>2<br>3<br>4<br>5<br>6	94<br>94<br>62<br>72<br>72<br>4	2 min<br>15 s<br>20 s<br>10 s<br>10 min<br>-	Steps 2-4 repeated 30 times
E-cadherin	1<br>2<br>3<br>4<br>5<br>6	94<br>94<br>62<br>72<br>72<br>4	2 min<br>15 s<br>20 s<br>10 s<br>10 min<br>-	Steps 2-4 repeated 25 times
Beta-actin	1<br>2<br>3<br>4<br>5<br>6	94<br>94<br>60<br>72<br>72<br>4	2 min<br>15 s<br>20 s<br>10 s<br>10 min<br>-	Steps 2-4 repeated 22 times

Table S10. Primers for qRT-PCR

Primer Name	Gene Name	Accession Nr.	Sequence	Annealing Temp. °C	Length bp
q-ve-cad cdh-5-fw<br>q-ve-cad cdh-5-rev	cdh-5	NM_001795.4	CACTTCCCCATCATGTAGGCAA<br>TTTTCGCCAGTGTCCTTGTCC	61,5	202
q-n-cad cdh-2-fw<br>q-n-cad cdh-2-rev	cdh-2	NM_001792.4	GAGTTTACTGCCATGACGTT<br>CTGATTCTGTACACTGCGTTC	61,5	128
q-E-cad -for<br>q-E-cad -rev	cdh-1	NM_004360.4	ACAACAAGCCCGAATTCACC<br>ACATCATCGTCCGCGTCT	61,5	109
q-TbP-fw<br>q-TbP-rev	TbP	NM_003194.4	GCCGAATATAATCCCAAGCG<br>TGGACTGTTCTTCACTCTTGGC	61,5	124
q-EF-2-fw<br>q-EF-2-rev	EF-2	NM_001961.3	GGTGGTCGGTGGCATCTAC<br>GTTGGACCTCAGGTCAGCG	61,5	148
q-HPRT-fw<br>q-HPRT-rev	HPRT	NM_000194.2	TTGCGACCTTGACCATCTTTG<br>CTTTGCTGACCTGCTGGATTAC	61,5	263

Table S11. qRT-PCR Program

Step	temp [°C]	time [s]	Number of Cycles
1	95	2 min	
2	95	15	
3	61,5	30	
4	72	10	2-4: 40x
5	72	2 min	
6	65-95	5	
7	20	~	

Table S12. Primary and secondary antibodies used for immunofluorescence staining

	Primary antibodies	Dilution of primary antibodies	Secondary antibodies	Dilution of secondary antibodies
VE-cadherin	VE-cadherin F8 mouse monoclonal IgG (Santa Cruz)	1:100	Alexa Fluor 568 dye (Thermo Fisher) Anti mouse	1:200
N-cadherin	Purified mouse anti-N-cadherin (BD Transduction Laboratories)	1:100	Alexa Fluor 568 dye (Thermo Fisher) Anti mouse	1:200
E-cadherin	E-cadherin rabbit antibody EP700Y (NOVUS Biologicals)	1:100	Alexa Flour 568 dye (Thermo Fisher) Anti rabbit	1:200
